# Improvement in renal function and its impact on survival in patients with newly diagnosed multiple myeloma

**DOI:** 10.1038/bcj.2015.20

**Published:** 2015-03-20

**Authors:** W I Gonsalves, N Leung, S V Rajkumar, A Dispenzieri, M Q Lacy, S R Hayman, F K Buadi, D Dingli, P Kapoor, R S Go, Y Lin, S J Russell, J A Lust, S Zeldenrust, R A Kyle, M A Gertz, S K Kumar

**Affiliations:** 1 Divisions of Hematology and Nephrology, Department of Internal Medicine, Mayo Clinic, Rochester, MN, USA

## Abstract

Renal impairment (RI) is seen in over a quarter of patients with newly diagnosed multiple myeloma (NDMM). It is not clear if reversal of RI improves the outcome to that expected for NDMM patients without RI. We evaluated 1135 consecutive patients with NDMM seen at the Mayo Clinic between January 2003 and December 2012. RI was defined as having a creatinine clearance (CrCl) <40ml/min. The median overall survival (OS) for patients with RI at diagnosis receiving and not receiving novel agent induction therapy was not reached vs 46 months (*P*<0.001). The median OS for patients with CrCl ⩾40 ml/min at diagnosis, CrCl <40 ml/min at diagnosis but improved to ⩾40 ml/min and CrCl <40 ml/min at diagnosis and remained <40 ml/min, were 112, 56 and 33 months, respectively (*P*<0.001). The complete renal response rate for patients with RI at diagnosis receiving novel agent induction therapy compared to the rest was 40 vs 16% (*P*<0.001). In conclusion, patients with reversal of RI have improved outcomes, but it remains inferior to patients with normal renal function at diagnosis. These results have implications for identifying early treatment strategies for patients at risk of developing renal insufficiency.

## Introduction

Multiple myeloma (MM) is the second most common hematologic malignancy among adults in the United States.^1^ Renal impairment (RI) is relatively common in patients with newly diagnosed MM (NDMM), ~20–40%,^2, 3^ and forms one of the defining features for diagnosis of symptomatic disease.^4^ RI in NDMM patients is often multifactorial and can be secondary to any of the following disease-related factors such as cast nephropathy, hypercalcemia, hyperuricemia, coexistent amyloidosis, light-chain deposition disease and so on.^5, 6^ RI is associated with a higher rate of treatment-related toxicity, early mortality and reduced overall survival (OS), especially when renal failure is advanced and dialysis support is required.^7, 8, 9, 10^ The effective management of RI associated with MM requires the prompt institution of anti-myeloma therapy and supportive measures.^11^

The incorporation of novel therapeutic agents such as immunomodulators (thalidomide, lenalidomide and pomalidomide) and proteasome inhibitors (bortezomib and carfilzomib) as well as improved supportive care options have led to significant improvements in the OS of patients with MM.^12, 13, 14, 15, 16^ These beneficial effects have also been described in newly diagnosed MM patients with RI.^17, 18, 19^ Although it has been shown that improvement in renal function can lead to improved survival in patients with MM,^19, 20^ it is not clear whether complete recovery of renal function improves survival outcomes to that experienced by MM patients who did not have RI at diagnosis. Thus, we addressed this question in a large cohort of patients with NDMM seen over the last decade at a single institution.

## Patients and methods

We identified NDMM patients seen at the Mayo Clinic, Rochester between 1 January 2003 and 31 December 2010 within 90 days of their diagnosis. Patients with light-chain amyloidosis with organ involvement confirmed via tissue biopsy were excluded from the current study. Approval for this study was obtained from the Mayo Clinic Institutional Review Board in accordance with the federal regulations and the principles of the Declaration of Helsinki.

Host and disease variables at diagnosis that were examined for prognostic significance included age, bone marrow plasma cell percentage, molecular cytogenetics status by *fluorescent in situ hybridization* (FISH), stage based on international staging system classification,^21^ plasma cell labeling index, serum monoclonal protein spike, urine monoclonal protein spike, hemoglobin, serum creatinine and lactate dehydrogenase (LDH). Levels >192 IU/dl for LDH were considered elevated. Patients who had a FISH analysis performed on their plasma cells were categorized as having high-risk disease if any of the following abnormalities: t(4;14), t(14;16) or t(14;20) were present at any time during their disease course, or a deletion 17p within 30 days of the diagnosis or any time before the diagnosis.^22, 23^ The initial treatment regimen used for induction was recorded and drugs such as thalidomide, lenalidomide and bortezomib were categorized as novel agents.

Serum creatinine at diagnosis and at last follow-up was obtained from clinical records and the creatinine clearance (CrCl) was calculated by the modification of diet in renal disease (MDRD) equation using the simplified four-variable MDRD formula: glomerular filtration rate=186.3 × (serum creatinine)^−1.154^ x (age in years)^−0.203^ × 1.212 (if patient is black) x 0.742 (if female).^24^ RI in NDMM patients was defined as an estimated glomerular filtration rate (eGFR)<40 ml/min/1.73 m^2^. For the analyses in this study, patients were categorized based on their renal function at diagnosis and response to therapy: group 1: CrCl⩾40 at diagnosis, group 2: CrCl<40 at diagnosis but improved to ⩾40 after therapy and group 3: CrCl<40 at diagnosis and remained <40 after therapy. The degree of restoration of renal function was evaluated according to the International Myeloma Working Group (IMWG) criteria, which considered renal complete response as a sustained increase in baseline eGFR to ⩾60 ml/min.^11^ Renal partial response was defined as an increase of eGFR from <15–30–59 ml/min and renal minor response as sustained improvement of baseline eGFR of <15 ml/min to 15–29 ml/min or if baseline eGFR was 15–29 ml/min, improvement to 30–59 ml/min. Early mortality was defined as death within 6 months of diagnosis. The primary end point of this study was OS, which was defined as the time from diagnosis to death with patients alive at the time of last follow-up censored at that date. The secondary end points were rate of early mortality and renal response in patients with RI.

The Fisher's exact test was used to assess for differences in nominal variables. Differences in continuous variables were compared using the Wilcoxon signed-rank test. Cox proportional hazard analysis was used to identify factors that were prognostic for reversal of RI and OS. Survival curves were constructed according to the Kaplan-Meier method and the curves were compared using log-rank test. All analyses were performed using JMP 10.0 (SAS Institute Inc., Cary, NC, USA).

## Results

The study included 1135 patients with NDMM seen between 1 January 2003 and 31 December 2010. The characteristics of these patients are described in Table 1. The median age at diagnosis was 65 years (range 22–93); 682 (60%) were male. The median estimated follow-up for the entire group from diagnosis was 76 months (95% confidence interval (CI) 72–79) and 515 (45%) patients had died at the time of this analysis.

### Baseline renal function and relationship with clinical features

The median creatinine at diagnosis was 1.1 mg/dl (range 0.4 to 11) with 124 (11%) patients presenting with a creatinine over 2 mg/dL. The median CrCl was 67 ml/min (range 4–219 ml/min) with 690 (61%), 322 (28%) and 123 (11%) of the patients with CrCl⩾Â60 ml/min, 30–59 ml/min and <30 ml/min, respectively. Comparison of baseline patient and disease-related features between patients with RI (CrCl<40 ml/min; *N*=192, 17%) vs those with CrCl⩾40 ml/min (*N*=943, 83%) are listed in Table 1. Patients with RI tended to be older (*P*<0.001), female (*P*=0.024), were more likely to have light-chain-only disease (*P*<0.001) and have a higher tumor burden (that is, international staging system 3; *P*<0.001), plasma cell labeling index (*P*=0.011), LDH (*P*<0.001) and bone marrow plasma cell percentage (*P*<0.001). There were 763 (67%) patients who received one or more of the novel agents as part of their initial therapy, of which 109 (14%) patients had a CrCl<40 ml/min at baseline.

### Impact of renal function at diagnosis on survival and early mortality

**Figure 1:**
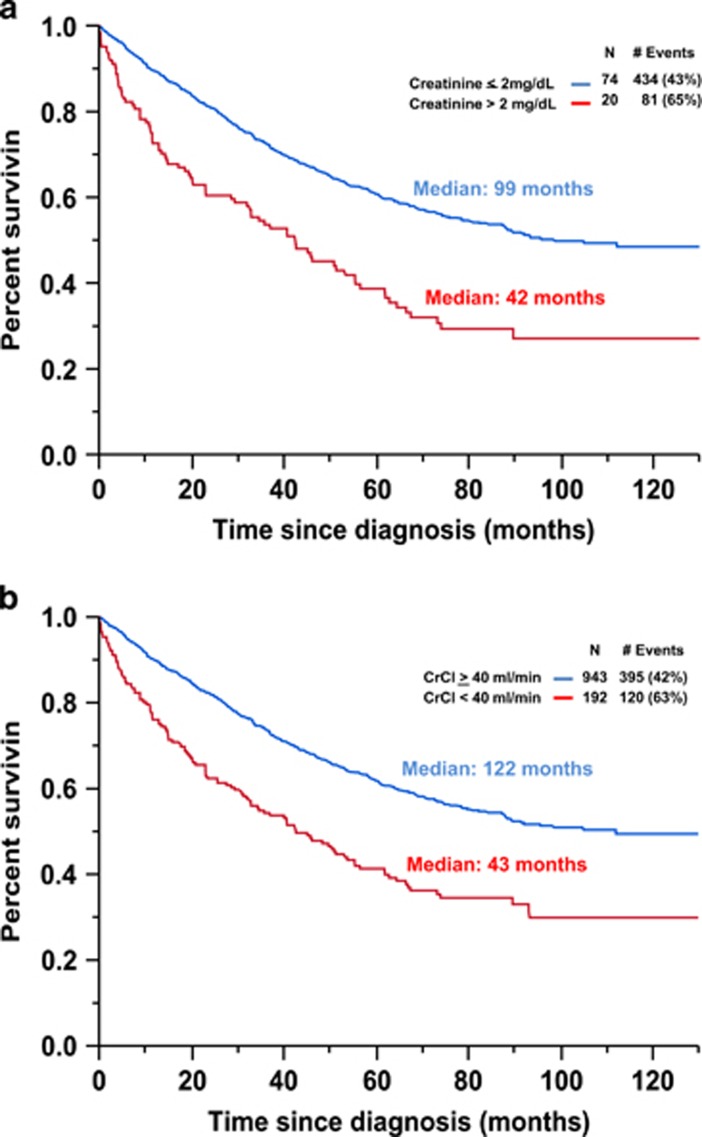
(**a**) Kaplan-Meier plot comparing overall survival between patients based on the presence or absence of a creatinine >2 mg/dl at diagnosis. (**b**) Kaplan-Meier plot comparing overall survival between patients based on the presence of an estimated creatinine clearance ⩾40 ml/min at diagnosis.

The median OS of the entire group of patients was 89 months (95% CI 74–112). The median OS from diagnosis for those with a creatinine >2 mg/dl was 42 months (95% CI 29–55) compared with 99 months (95% CI 87 to not reached) for the rest; (*P*<0.001; Figure 1a). The median OS (95% CI) from diagnosis among patients with CrCl⩾40 ml/min and <40 ml/min were 112 (88 to not reached) and 43 months (33–55), respectively (*P*<0.001; Figure 1b). Of the 192 patients with CrCl<40 ml/min, 32 (16%) required dialysis at diagnosis. The OS of patients requiring dialysis compared to those with CrCl<40 ml/min not requiring dialysis was 45% vs 31% (*P*=0.58). The rate of early mortality among patients with CrCl⩾40 ml/min and <40 ml/min were 6 and 16%, respectively (*P*<0.001).

**Figure 2:**
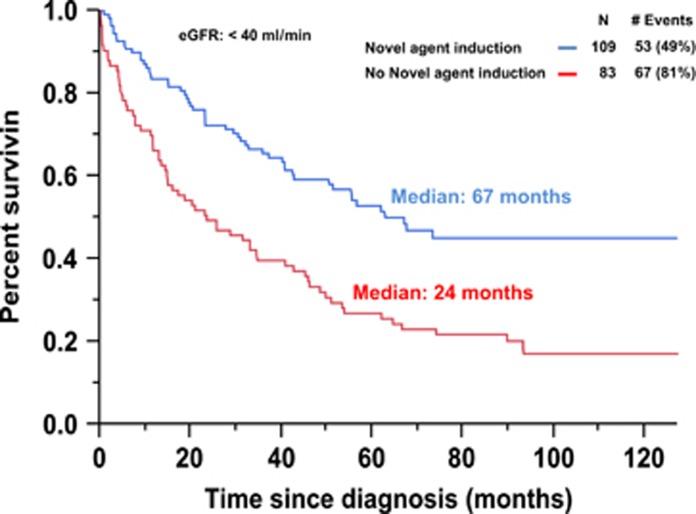
Kaplan-Meier plot comparing overall survival between patients with an estimated creatinine clearance of <40 ml/min based on the presence or absence of a novel agent induction regimen.

Among the patients with CrCl<40 ml/min at diagnosis, the median OS from diagnosis for those receiving a novel agent was 67 months compared with 24 months for those not receiving a novel agent-based induction (*P*<0.001; Figure 2). The rate of early mortality among patients with CrCl<40 ml/min at diagnosis receiving a novel agent was 10% compared with 24% for those not receiving a novel agent-based induction regimen (*P*=0.011).

### Improvement in renal function and its relationship with survival and early mortality

Of the 192 patients with a CrCl<40 ml/min at diagnosis, any improvement in CrCl was seen in 152 (82%) patients. The median time to the best CrCl was 4 months (range <1–34 months) from diagnosis. Only the absence of light-chain MM predicted for recovery of renal function to a CrCl to ⩾40 ml/min in a proportional hazards model (hazard ratio 1.96, 95% CI 1.27–3.14; *P*=0.002).

Upon applying the IMWG criteria for renal response to the 192 patients with a CrCl<40 ml/min at diagnosis, 56 (29%) had a complete response, 16 (8%) had a partial response, 51 (27%) had a minimal response and 69 (36%) had an not reached. The renal complete response rate for patients with CrCl<40 ml/min at diagnosis receiving novel agent induction therapy compared to those not receiving novel agent induction therapy was 40 vs 16% (*P*<0.001). The renal complete response rate in patients with CrCl<40 ml/min at diagnosis was higher for those receiving bortezomib-based induction therapies compared to those receiving immunomodulator-based induction therapies, however, this was not statistically significant (45 vs 28% *P*=0.09).

**Figure 3:**
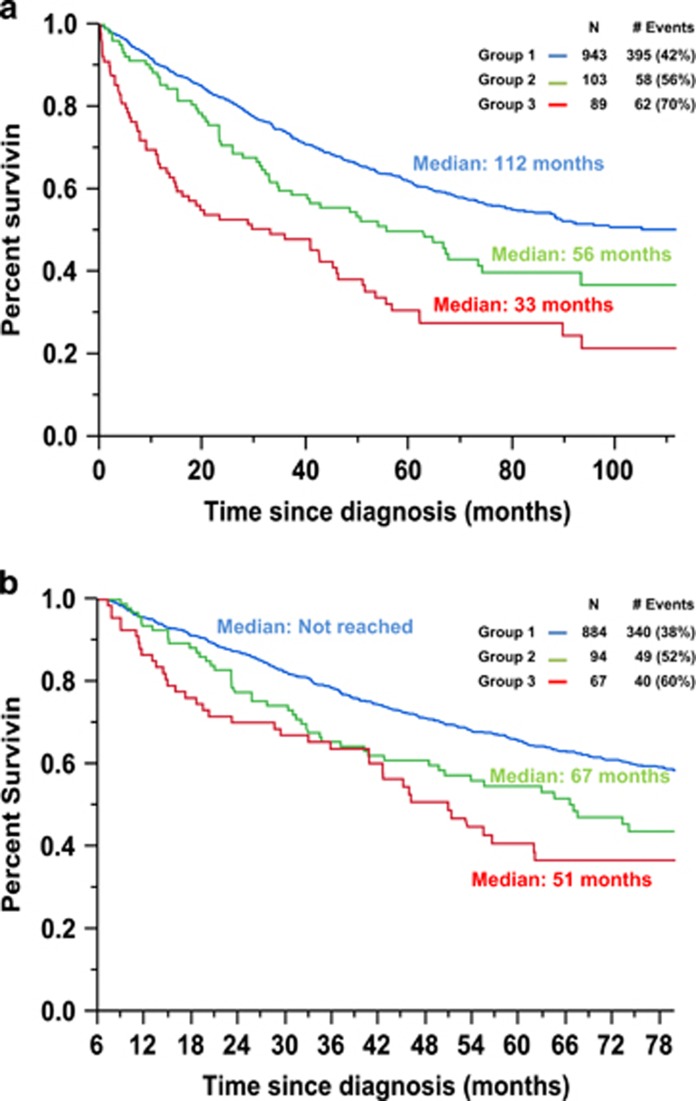
(**a**) Kaplan-Meier plot comparing overall survival between groups 1, 2, and 3 based on their renal function at diagnosis and response to therapy: group 1, CrCl⩾40 at diagnosis; group 2, CrCl<40 at diagnosis but improved to ⩾40 after therapy; and group 3, CrCl<40 at diagnosis and remained <40 after therapy. (**b**) Kaplan-Meier plot comparing overall survival at a 6-month landmark based on their renal function at diagnosis and response to therapy: group 1, CrCl⩾40 at diagnosis; group 2, CrCl<40 at diagnosis but improved to ⩾40 after therapy; and group 3, CrCl<40 at diagnosis and remained <40 after therapy.

Upon categorizations based on the renal response to therapy as described in the methods, patients in group 2 (*N*=103, 9%) had a median OS of 56 months compared with 33 months for patients in group 3 (*N*=89, 8% *P*=0.006), but this was still significantly lower than the median OS for patients in group 1 (*N*=943, 83%), which was 112 months (*P*<0.001; Figure 3a). A landmark analysis at 6 months, to allow for sufficient duration of therapy, revealed a median OS from diagnosis of not reached, 67 months and 51 months for patients in groups 1, 2 and 3, respectively (*P*=0.175 between groups 2 and 3; *P*=0.007 between groups 1 and 2; Figure 3b).

### Improvement in renal function and its relationship with early mortality

An analysis of early mortality (death within 6 months of diagnosis) between the three groups was performed. The rates of early mortality were 6, 9 and 25% for groups 1, 2 and 3, respectively (*P*=0.003 between groups 2 and 3; *P*=0.298 between groups 1 and 2). When the analysis was restricted to only patients who received novel agent induction therapy, the rate of early mortality among the NDMM patients with RI was not statistically different between those who received bortezomib-based induction and immunomodulator-based induction with lenalidomide or thalidomide (9 vs 11%, *P*=0.100). We then examined the last available creatinine in each of the patients. When evaluated over the course of their disease, patients in group 2 were more likely to eventually worsen their renal function again leading to RI toward the end of their follow-up in this study compared to patients in group 1 (34 vs 9% *P*<0.001).

### Predictors of worse OS at diagnosis

In a univariable analysis assessing predictors for OS, CrCl<40 ml/min, no novel agent induction therapy, age ⩾70, international staging system stage 3, high-risk FISH and LDH>192 IU/dl were all found to predict for worse OS; however, only age ⩾70 (*P*<0.001), high-risk FISH (*P*<0.001) and lack of novel agent induction therapy (*P*=0.008) retained their negative prognostic significance in a multivariable analysis (Table 2). Though plasma cell labeling index was found to be significant in the univariable analysis predicting for OS, it was excluded from the multivariable analysis due to more than half of the patients not having it performed.

## Discussion

The current IMWG definition of MM-related RI requires a serum creatinine of 2 g/dl or higher that is unexplainable by any other etiology.^25^ However, this cutoff may fall short in identifying all NDMM patients with RI since the serum creatinine can be influenced by factors such as muscle mass. Conversely, a cutoff of CrCl <60 ml/min may inappropriately consider older NDMM patients with age-related decline in their CrCl as having MM-related RI. In this study, we used a CrCl cutoff of <40 ml/min as it has appeared to be optimal in identifying NDMM patients with RI when compared to the previously mentioned cutoffs.^26^

By using our CrCl cutoff of 40 ml/ml, RI at diagnosis was present in almost 20% of the patients. Of these patients, more than half of them (54%) had reversal of their RI upon institution of anti-myeloma induction therapy. This study demonstrated an improvement in survival among NDMM patients with RI as a result of novel agent therapy as seen in Figure 2. Furthermore this study suggests that though RI in NDMM patients is associated with a worse OS as well as higher rates of early mortality, it is not an independent predictor of worse OS as seen in the multivariable model (Table 2). However, this study demonstrates that even if NDMM patients with RI experience a resolution of their RI upon receiving myeloma directed therapy (group 2), they do not have equivalent survival outcomes to those NDMM patients without RI (group 1) as seen in Figure 3a. Furthermore, even after accounting for early mortality by performing a landmark analysis at 6 months, patients in group 2 still do not have equivalent survival outcomes as those patients in group 1 (Figure 2). However, reversal of RI is still important to achieve in NDMM patients. This is because patients in group 3 who have RI at diagnosis but never recover their renal function have a worse median OS to patients in group 2 (33 vs 56 months, *P*=0.006). This survival benefit of reversal of RI seen in group 2 is observed likely due to their significantly lower rate of early mortality in comparison to group 3 (8 vs 17% *P*=0.004). When a landmark analysis was performed at 6 months, the median OS for patients in group 2 compared to group 3 was 67 vs 51 months (*P*=0.175).

Our study also evaluated the renal function of all the patients at their last follow-up. We observed that even though patients in group 2 had a reversal of their RI, they were more likely to eventually re-worsen their renal function and experience RI again compared to the patients in group 1 (34 vs 9% *P*<0.001); this suggests that NDMM patients with RI at diagnosis likely have a higher propensity for RI during their disease course compared to their NDMM counterparts who do not have RI at diagnosis.

Several studies have confirmed the beneficial effect of novel agent induction therapy in NDMM patients with RI in comparison to conventional chemotherapy.^27, 28^ This is likely because novel agents have been associated with improved depth of paraprotein response in myeloma and this likely translates to higher rates of improvement in renal function.^29, 30, 31, 32^ RI at diagnoses has not been found to weaken the responses provided by novel agent induction therapy.^33^ Furthermore, unlike most conventional chemotherapeutic agents, novel agents such as bortezomib and thalidomide have safe pharmacokinetic and pharmacodynamic properties in the setting of severe RI;^34^ lenalidomide, even though it is mainly renally excreted, can also be safely used if appropriate dose modifications are implemented and close evaluation for toxicities are undertaken in patients with mild to moderate RI.^35^ Novel agents such as bortezomib have also been reported to have protective effects in the renal tubular cells^36^ and inhibitory effects on the pro-inflammatory and fibrotic pathways within the renal microenvironment^37^ in addition to its previously described anti-myeloma activity.

However, despite the survival gains experienced with the emergence of novel agents in the upfront management of MM patients with RI at diagnosis, there is still room for improvement. Acute renal failure has been a major cause of early mortality in previous observational studies involving NDMM patients.^9, 38^ Even in this study, we observed a 16% early mortality rate in NDMM patients with CrCl<40 ml/min treated with a novel agent induction regimen; this signifies the need for earlier and more effective interventions in this subgroup of patients.

Our study also indicated that certain patient and disease-related characteristics predicted for the presence of RI at diagnosis in NDMM patients. Age >70 years was the only patient characteristic associated with RI at diagnosis. However, higher disease burden as suggested by an international staging system 3, high-risk cytogenetics by FISH, light-chain-secreting-only disease, higher bone marrow plasma cell percentage and higher LDH were all determinants of plasma cell biology that were associated with RI at diagnosis. In contrast, only NDMM patients who did not have light-chain-secreting-only disease were most likely to recover their renal function. In this study, older age, high-risk FISH, elevated LDH, high plasma cell labeling index and lack of novel agent use during induction were independent predictors of worse OS. This suggests that RI at diagnosis may not be an adverse prognostic marker in NDMM patients. In a study by Eleftherakis-Papapiakovou *et al.,*^19^ RI was also not found to be independently associated with inferior survival likely as a result of novel agent use during induction therapy.

There are several limitations to this study. First, the etiology of RI in each patient was not included in the analyses. Given the advanced age of the average NDMM patient, it is possible that their RI can also be associated with a decline in renal function as a result of other medical comorbidities such as diabetes, hypertension, vascular disease, drug-induced issues and so on. unrelated to the MM. Furthermore, a renal biopsy is not necessarily required or performed in every NDMM patient with RI^11^ and we do not have that information on all of our patients making it difficult to know the true etiology of RI in our patient population. Second, we are unable to determine with certainty whether certain novel agents used during induction such as bortezomib is superior to immunomodulators like thalidomide or lenalidomide in this subpopulation with RI due to the potential bias in patient selection for certain therapies and the high likelihood of most patients receiving bortezomib in the salvage setting. Third, the retrospective nature of this study prevents us from truly understanding the etiology of early mortality in these patients, that is, disease-related morbidity vs therapy-related toxicity leading to early mortality. Also in patients with acute renal impairment, the traditional equations to calculate glomerular filtration rate such as the MDRD formula do not provide accurate assessments of their true CrCl.^39^ Nevertheless, the MDRD equation has been adopted by the IMWG in monitoring renal function and response in patients with newly diagnosed MM. ^11^

Data from randomized control trials comparing different induction therapy regimens in NDMM patients with RI are sparse. Nevertheless, our data confirm the improvement in renal response that novel agents have had on NDMM patients with RI. They have also decreased early mortality in these patients as well as improved OS. However, reversal of RI alone does not appear to elevate the expected OS of such NDMM patients with RI at diagnosis to that of patients without RI at diagnosis. The findings from this study imply the need for instituting early treatment strategies in order to prevent patients from developing RI. For example, redefining the existing CRAB criteria required to diagnose MM by including criteria such as free light chain ratio >100 (ref. 40) or a bone marrow plasma cell percentage of 60 or higher^41^ may identify NDMM patients requiring therapy prior to them developing RI. In addition, for those NDMM patients already with RI, further work is required to determine the optimal management so as to continue to reduce associated morbidity and mortality.

## Figures and Tables

**Table 1:** Clinical and laboratory characteristics of 1135 NDMM patients based on the presence or absence of RI

*Clinical feature*	*All patients (*N=*1135)*	*Pts with RI (*N=*192)*	*Pts without RI (*N=*943)*	P-*value*
Age (years)	65 (22–93)	69 (29–92)	64 (22–93)	*<0.001*
*ISS, stage (*N *(%))*				*<0.001*
** **I	256 (25)	3 (2)	253 (30)	
** **II	466 (45)	27 (16)	439 (51)	
** **III	303 (30)	141 (82)	162 (19)	
Male (*N* (%))	682 (60)	101 (53)	581 (62)	*0.024*
Hemoglobin (gm/dl)	11.2 (5.7–17.2)	9.9 (6.5–16.5)	11.4 (5.7–17.2)	*<0.001*
Creatinine (mg/dl)	1.1 (0.4–11)	2.6 (1.4–11)	1.0 (0.4–1.8)	*<0.001*
Requiring dialysis at diagnosis (*N* (%))	32 (3)	32 (16)	0	*<0.001*
Calcium (mg/dl)	9.6 (2.4–16.5)	9.4 (6.6–16.5)	9.6 (2.4–15.3)	0.162
β_2_ Microglobulin (mg/dl)	3.8 (0.7–88.4)	10.4 (2.14–88.4)	3.35 (0.74–77.5)	*<0.001*
Elevated LDH (>192 IU/dl, %)	245 (31%)	65 (48%)	180 (28%)	*<0.001*
Serum albumin (g/dl)	3.5 (1.9–5.8)	3.3 (2.0–4.4)	3.5 (1.9–5.8)	*<0.001*
PCLI>1% (*N* (%))	228 (36)	50 (47)	178 (34)	*0.011*
Bone marrow PC%	50 (0–100)	60 (0–100)	50 (0–100)	*<0.001*
dFLC (mg/dl)	43 (0–7949)	210 (0–7949)	30 (0–2199)	*<0.001*
Urine M spike (g/dl)	0.29 (0–16.35)	1.16 (0–10.1)	0.18 (0–16.35)	*<0.001*
Urine albumin (g/dl)	0.06 (0–6.78)	0.19 (0–6.62)	0.04 (0–6.78)	*<0.001*
Light chain MM (*N* (%))	198 (18)	62 (32)	136 (14)	*<0.001*
Received novel agent (*N* (%))	763 (67)	109 (57)	654 (69)	*0.001*
** **Thalidomide (*N* (%))	171 (15)	35 (32)	136 (20)	
** **Lenalidomide (*N* (%))	464 (41)	29 (27)	435 (67)	
** **Bortezomib (*N* (%))	128 (11)	45 (41)	83 (13)	
Underwent ASCT (*N* (%))	471 (42)	61 (32)	410 (43)	*0.003*
High-risk FISH (661 pts; *N* (%))	152 (23)	21 (25)	131 (23)	0.680
** **t(4;14); *N* (%)	57 (9)	6 (7)	51 (9)	
** **t(14;16); *N* (%)	31 (5)	7 (8)	24 (4)	
** **t(14;20); *N* (%)	1 (<1)	1 (1)	0 (0)	
** **Deletion 17p (*N* (%))	88 (13)	11 (13)	77 (13)	

Abbreviations: ASCT, autologous stem cell transplant; dFLC, FLC difference; FISH, fluorescent *in situ* hybridization; FLC, free light chain; ISS, international staging system; LDH, lactate dehydrogenase; M spike, monoclonal protein spike; NDMM, newly diagnosed multiple myeloma; PC%, plasma cell percentage; PCLI, plasma cell labeling index; Pts, patients; RI, renal impairment. Italics signifies statistically significant (*P*<0.05).

**Table 2:** Multivariable analysis of clinical and laboratory factors at diagnosis associated with OS

*Variables at diagnosis*	*Univariate analysis*		*Multivariate analysis*	
	*Hazard ratio (95% CI)*	P-*value*	*Hazard ratio (95% CI)*	P-*value*
Age ⩾70	*2.32 (1.95–2.76)*	*<0.001*	*2.48 (1.77–3.44)*	*<0.001*
ISS 3	*2.12 (1.75–2.56)*	*<0.001*	1.35 (0.90–1.99)	0.139
LDH>192 IU/dL	*1.43 (1.16–1.76)*	*<0.001*	1.33 (0.94–1.88)	0.106
Novel agent induction	*0.47 (0.40–0.56)*	*<0.001*	*0.63 (0.46–0.89)*	*0.008*
High-risk FISH	*1.89 (1.43–2.48)*	*<0.001*	*2.00 (1.41–2.80)*	*<0.001*
CrCl<40 ml/min (RI at diagnosis)	*1.90 (1.54–2.32)*	*<0.001*	1.21 (0.72–2.12)	0.477

Abbreviations: CI, confidence interval; CrCl, creatinine clearance; FISH, fluorescent *in situ* hybridization; ISS, international staging system; LDH, lactate dehydrogenase; OS, overall survival; RI, renal impairment. Italics signifies statistically significant (*P*<0.05 in the Univariate analysis and *P*<0.008 in the Multivariate analysis).
